# Van der Knaap Disease Unveiled: A Radiological-Genomic Journey From Seizure to Diagnosis

**DOI:** 10.7759/cureus.112696

**Published:** 2026-07-15

**Authors:** Manjari Jaiswal, Arushi Yadav, B R Goyal, Archana Mathur, Gyan Chand

**Affiliations:** 1 Radiology, Max Super Speciality Hospital, Patparganj, New Delhi, IND

**Keywords:** megalencephalic leukoencephalopathy, mlc1, neuroimaging, subcortical temporal cysts, van der knaap

## Abstract

Megalencephalic leukoencephalopathy with subcortical cysts (MLC) is a rare autosomal recessive leukodystrophy predominantly caused by mutations in the MLC1 gene, characterized by early-onset macrocephaly, progressive neurological deterioration, and distinctive neuroimaging findings. We report this case to highlight a late-onset seizure presentation in a 12-year-old boy with MLC and significant clinico-radiological dissociation. While the patient exhibited pathognomonic MRI findings, including extensive white matter fluid-attenuated inversion recovery (FLAIR) hyperintensity and characteristic subcortical cysts in anterior temporal and occipitoparietal regions, he maintained independent ambulation, which is in striking contrast to the severity of the imaging findings. The corpus callosum, internal capsule, and posterior fossa structures were unaffected. Vasogenic edema was evidenced by elevated apparent diffusion coefficient (ADC) values without diffusion restriction. Whole exome sequencing identified a homozygous frameshift duplication mutation (c.135dup; p.Cys46Leufs*34) in the MLC1 gene, confirming the diagnosis of classical MLC1-related disease. This report underscores the necessity of integrated radiological-genomic assessment to differentiate MLC from other macrocephalic leukodystrophies to enable early diagnosis and initiate appropriate supportive care and genetic counseling. It aids early screening of close family members, facilitating genetic counseling and informed reproductive decision-making for affected families, thereby decreasing the chances of further transfer of the mutant gene in subsequent generations.

## Introduction

Megalencephalic leukoencephalopathy with subcortical cysts (MLC), or Van der Knaap disease, was first described by Van der Knaap et al. in 1995, who reported a cohort of eight children presenting with leukoencephalopathy, white matter swelling, and a discrepantly mild clinical course, thereby delineating this as a distinct clinico-radiological entity [1]. Subsequently, Singhal et al. described megalencephalic leukodystrophy in 26 patients from the Agarwal community of India, identifying significant familial clustering and confirming the c.135dup MLC1 mutation as a prevalent founder mutation within this community [2]. The disease is primarily caused by mutations in the MLC1 or HEPACAM genes [3,4], which disrupt astrocytic regulation of brain ion and water homeostasis, leading to chronic intramyelinic edema and characteristic white matter swelling [5].

It is classically characterized by infantile-onset macrocephaly and progressive motor deterioration with a discrepantly mild clinical course relative to radiological severity. Phenotypic variability exists with atypical presentations occasionally reported [1]. This clinical heterogeneity necessitates reliance on pathognomonic neuroimaging findings for confident diagnosis. MRI characteristically reveals diffuse white matter swelling on T2/fluid-attenuated inversion recovery (FLAIR) sequences, anterolateral temporal subcortical cysts, and facilitated diffusion on diffusion-weighted (DW) and apparent diffusion coefficient (ADC) images [1]. Selective preservation of the corpus callosum, internal capsule, and posterior fossa structures helps rule out other differentials [1].

Although MLC classically manifests with progressive pyramidal dysfunction and cerebellar ataxia, our case was unique in that it presented with late-onset generalized tonic-clonic seizures at 12 years of age. On imaging, there was an exceptionally large (~8.0 cm) occipito-parietal subcortical cyst besides the characteristic imaging features of MLC. A cyst of this size coexisting with preserved ambulation and relatively mild cognitive decline has not been reported in the current literature, to the best of our knowledge. Thus, this case highlights the importance of recognizing atypical radiological and phenotypic presentations of rare leukodystrophies, thereby contributing valuable evidence to the evolving literature on phenotypic variability in MLC.

## Case presentation

A 12-year-old boy presented to our emergency department following an episode of generalized tonic-clonic seizure with a two-year history of progressive cognitive decline. Motor development had been delayed since early childhood. The patient achieved independent walking at two years of age, which was delayed compared to the normal milestone of 1 to 1.5 years. Despite achieving independent ambulation, the child never developed the ability to run. At presentation, his gait was described as unsteady with frequent stumbling and falls. No history of consanguinity was present. Examination revealed macrocephaly (58.0 cm head circumference, >97th percentile and >4 SD). However, the parents were unable to recognize the significance of the increasing head circumference and considered it to be a normal phenomenon in their community.

Routine blood biochemistry, liver and renal function tests, serum lactate, and thyroid function tests were within normal limits. Metabolic screening and CSF analysis showed normal patterns. Electroencephalography (EEG) demonstrated generalized background suppression with marked temporal predominance, likely secondary to extensive white matter disease. No epileptiform discharges were captured.

**Figure 1:**
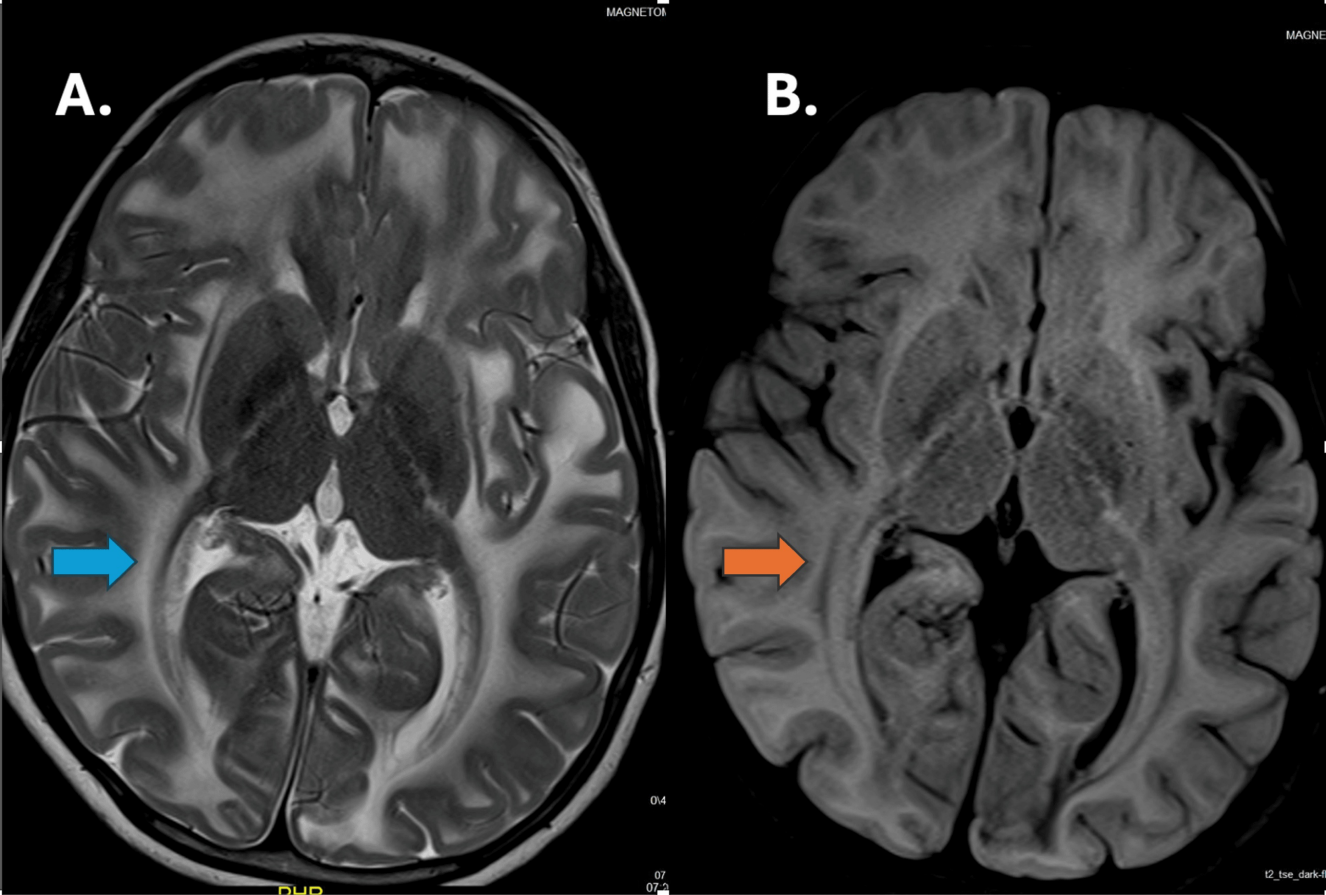
(A) Axial T2W 3-T and (B) axial FLAIR 3-T MRI images demonstrating bilateral, diffuse and symmetrical supratentorial white matter hyperintensities consistent with white matter edema (blue and orange arrows). T2W, T2 weighted; FLAIR, fluid-attenuated inversion recovery

Brain MRI demonstrated diffuse, bilateral, symmetrical supratentorial white matter hyperintensity on T2-weighted and FLAIR sequences (Figures 1A, 1B).

**Figure 2:**
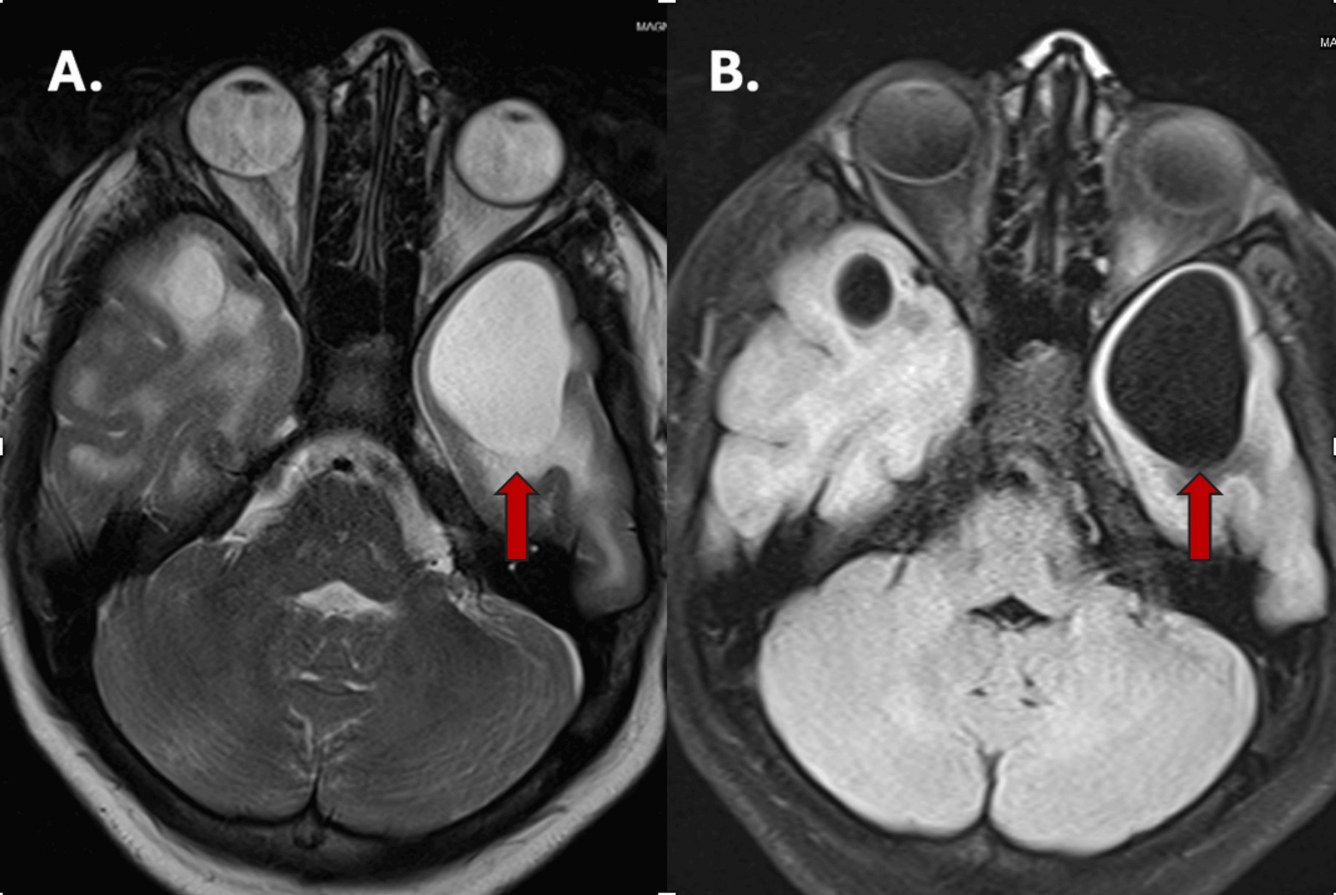
(A) Axial T2W 3-T and (B) axial FLAIR 3-T MRI images demonstrating classical subcortical temporal cysts (red arrows) showing complete suppression on FLAIR sequences. T2W, T2 weighted; FLAIR, fluid-attenuated inversion recovery

Well-demarcated subcortical cysts were localized to the anterior temporal regions (Figures 2A, 2B).

**Figure 3:**
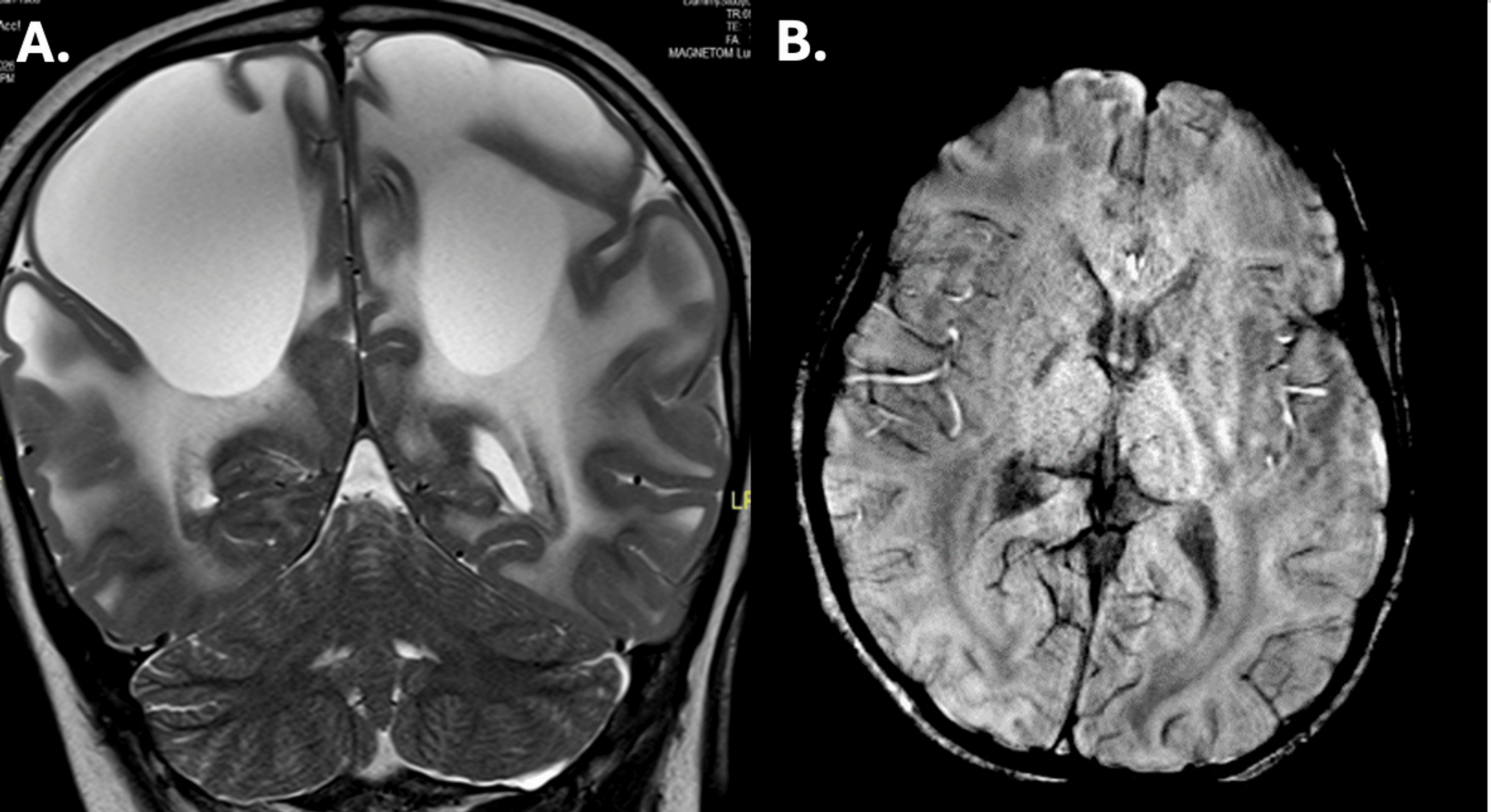
(A) Coronal T2W 3-T MRI image demonstrating the large occipito-parietal cysts with sparing of posterior fossa structures and (B) axial T2*GRE 3-T MRI image showing bilateral normal basal ganglia without calcifications. T2W, T2 weighted; GRE, gradient recalled echo

A significantly large cyst measuring 8.0 cm was identified in the right occipito-parietal region (Figure 3A). These cysts exhibited a CSF-like signal with FLAIR suppression. The corpus callosum, internal capsule, and posterior fossa structures showed relative preservation (Figure 3A). T2*GRE (gradient recalled echo) sequences confirmed the absence of basal ganglia calcification (Figure 3B).

**Figure 4:**
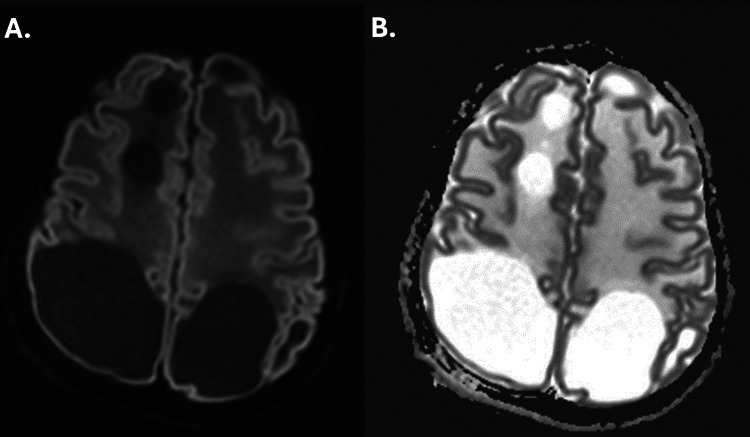
(A) Axial DWI 3-T and (B) axial ADC 3-T MRI images showing subcortical cysts in the bilateral occipitoparietal regions. No evidence of diffusion restriction is seen in the deep white matter anterior to the above-mentioned cysts, consistent with vasogenic edema. DWI, diffusion-weighted imaging; ADC, apparent diffusion coefficient

DW imaging (DWI) revealed increased ADC values (1.2-1.4 × 10⁻³ mm²/s) within the altered white matter, indicating vasogenic edema rather than cytotoxic injury (Figures 4A, 4B).

Genetic testing using the next-generation sequencing method revealed homozygous frameshift duplication variant c.135dup; p.Cys46Leufs*34, in the MLC1 gene on chromosomal position chr22:50084767:A>AG. This variant was present in 71 out of 72 reads at the locus. It was located in exon 2 of the transcript NM_015166.4, leading to protein truncation 34 amino acids downstream of codon 46. The total length of the wild-type protein was 377 amino acids. As per the American College of Medical Genetics and Genomics (ACMG) classification, the variant was classified as pathogenic with criteria PVS1, PM2, and RP5.

The patient was managed conservatively with anti-epileptics and physiotherapy and showed no clinical deterioration at the three-month follow-up. Further testing of the reported variant in parents and other affected or unaffected family members was recommended. However, the parents and the family members were not keen to do the same.

## Discussion

This case demonstrates MLC, which represents a distinct form of childhood-onset leukodystrophy with a characteristic clinical and radiological spectrum [1,2]. It is seen in consanguineous marriages in some families from the Agarwal community of India, as described by Singhal et al. in 26 patients from the same community in 1996 [2]. MLC1-related disease typically presents with classical features including infantile-onset macrocephaly, slowly progressive motor deterioration with ataxia and spasticity, and relatively mild cognitive impairment [1]. However, as demonstrated in our case, seizures and prominent cognitive decline can be presenting features, expanding the recognized phenotypic spectrum of the disease. Seizures usually present early in the course of the disease; however, in our case, there was a late presentation, at the age of 12 years.

The disease follows an autosomal recessive inheritance pattern and is primarily caused by mutations in two genes: MLC1 (encoding MLC1 protein), accounting for approximately 75% of cases [3], and HEPACAM (encoding GlialCAM protein), responsible for the remaining cases [4].

The MLC1 gene, located on chromosome 22q13.33, encodes a membrane protein predominantly expressed in astrocytic endfeet at the blood-brain barrier and brain-cerebrospinal fluid interfaces. MLC1 and GlialCAM proteins form a complex at astrocytic membranes that regulates volume-sensitive chloride channels and maintains fluid balance in the white matter [5].

The fundamental pathophysiological mechanism underlying MLC involves dysfunction of brain ion and water homeostasis, leading to chronic intramyelinic edema. Disruption of this system results in the accumulation of fluid within the myelin sheaths, causing the characteristic white matter swelling observed on neuroimaging.

Hamilton et al. conducted an international multicenter study documenting the clinical and radiological spectrum of MLC across both MLC1 and HEPACAM-related disease, establishing genotype-phenotype correlations and confirming that complete loss-of-function mutations such as c.135dup consistently produce the classical phenotype. No clinical or radiological differences were identified between MLC1 and MLC2A patients, while MLC2B patients demonstrated a consistently milder phenotype with preserved motor function, relatively frequent intellectual disability and autism, and radiological improvement on follow-up MRI. Early disease stage MRI features suggestive of MLC2B included absence of signal abnormalities in the posterior limb of the internal capsule and cerebellar white matter, with rarefied subcortical white matter rather than true subcortical cysts. Radiological improvement was also observed in two MLC1 patients, highlighting phenotypic variability even within the same genotypic subgroup [6]. The most comprehensive recent genetic analysis was published by Passchier et al. in 2024, documenting 508 patients with 151 unique variants in MLC1, 29 unique variants in GLIALCAM, 2 in GPRC5B, and 1 in AQP4, expanding the genetic landscape of MLC beyond classical genes and underscoring the evolving complexity of this disorder [7]. Consistent with these studies, genetic testing in our patient revealed a homozygous c.135dup mutation in the MLC1 gene, resulting in p.Cys46Leufs*34 frameshift mutation causing premature termination and a truncated non-functional protein. This mutation, particularly prevalent in specific Indian ethnic communities, suggesting a common ancestral origin, represents one such founder mutation predominantly identified within the Agarwal community [2,3]. Our patient also belonged to the subgroup of the same ethnic community.

Ganguly et al. further expanded the phenotypic spectrum of MLC, reporting seven cases that exhibited movement disorders beyond cerebellar ataxia, including dystonia and Parkinsonism. Six of them, belonging to the Agarwal community, had the common c.135dup variant, highlighting considerable phenotypic variability of this founder mutation [8]. Das and Dhibar conducted a 10-year case series of 12 patients with van der Knaap disease, reporting that seizures were present in 66.7% of cases, motor and intellectual impairment were more severe in patients with extra-temporal subcortical cysts, and that diffuse white matter involvement with anterior temporal subcortical cysts was observed universally across all cases, reinforcing the diagnostic importance of MRI in this condition [9]. Our case had both anterior temporal and extra-temporal subcortical cysts with diffuse white matter involvement; however, intellectual impairment was moderate, and the patient had preserved ambulation at the time of presentation. Pramod et al. presented a rare case of a six-year-old boy with unusual findings on MRI, including internal capsule, brainstem, and cerebellar involvement, subependymal heterotopia and cysts, and cortical laminar necrosis, along with typical findings of megalencephalic leukoencephalopathy and subcortical cysts [10].

Collectively, these studies reinforce that while the neuroimaging findings of MLC remain consistent, clinical manifestations can vary considerably, necessitating long-term surveillance and integrated radiological-genetic evaluation for definitive diagnosis and management.

The imaging spectrum in our case was virtually pathognomonic. Anterolateral temporal subcortical cysts represent the most specific feature, present in over 90% of MLC patients, and represent areas of severe myelin vacuolation and tissue rarefaction [11]. These cysts progressively enlarge over time. The exceptionally large occipito-parietal subcortical cyst with relatively preserved motor functions and maintained ambulation with absence of severe spasticity highlights the clinico-radiological dissociation described by van der Knaap et al., who characterized this classical MLC feature of a discrepantly milder clinical course relative to extensive imaging findings [1]. Selective preservation of corpus callosum, internal capsule, and posterior fossa structures is critical for diagnosis and reflects regional variations in myelin composition and astrocytic water transport mechanisms. The increased ADC values confirm vasogenic edema from intramyelinic fluid accumulation rather than cytotoxic injury.

Critical imaging features differentiating MLC from other macrocephalic leukodystrophies include selective preservation of corpus callosum, internal capsule, and posterior fossa structures reflecting regional variations in myelin composition (Table 1). Alexander disease was excluded due to the absence of its characteristic features, including frontal white matter predominance, involvement of caudate heads and thalami, periventricular rim enhancement, brainstem and dentate nuclei involvement, and contrast enhancement [12]. Canavan disease was excluded by a different white matter involvement pattern, as Canavan typically shows early diffuse U-fiber involvement with relative central white matter sparing and characteristic globus palladi involvement [13], with another distinctive feature being the presence of cytotoxic edema and diffusion restriction, features not present in our case [14]. Absence of basal ganglia calcification on T2-star sequences is essential for excluding Labrune syndrome, the primary differential diagnosis, which shows extensive bilateral basal ganglia and dentate calcifications as its pathognomonic feature [15].

**Table 1:** Radiological features differentiating MLC from its mimics MLC, megalencephalic leukoencephalopathy with subcortical cysts; DWI, diffusion-weighted imaging; ADC, apparent diffusion coefficient Table credits: Author Manjari Jaiswal Source: [7-9]

Feature	MLC (Van der Knaap)	Alexander Disease	Canavan Disease
Primary involvement	Diffuse supratentorial white matter	Frontal white matter predominance	Diffuse white matter with early U-fiber involvement
Subcortical cysts	Pathognomonic; anterior temporal and frontoparietal	Rare; typically periventricular if present	Not a characteristic feature
Sparing pattern	Corpus callosum and internal capsule preserved	Caudate heads, thalami, and brainstem involved	Relative central white matter sparing
Basal ganglia	Normal signal; no calcification	Significant involvement and swelling	Characteristic globus pallidus involvement
DWI/ADC findings	Vasogenic edema (increased ADC)	Variable; contrast enhancement often present	Cytotoxic edema (diffusion restriction)

Furthermore, no disease-modifying therapy exists. Management remains supportive with antiepileptics for seizures, physical and occupational therapy, cognitive support, and genetic counselling with 25% recurrence risk for future pregnancies. Genetic counselling was provided with recommendations for prenatal diagnosis options and extended family carrier screening.

## Conclusions

This case demonstrates features - anterolateral temporal subcortical cysts, diffuse white matter swelling, and macrocephaly - pathognomonic for MLC. However, cyst size does not predict clinical severity in MLC, as large cysts can coexist with preserved motor function, demonstrating characteristic clinico-radiological dissociation. The patient may present with seizures and cognitive decline years after initial macrocephaly and motor symptoms. The c.135dup MLC1 mutation is a founder mutation, commonly seen in the Agarwal community, enabling targeted genetic screening in at-risk populations. Appropriate diagnosis, based on clinical characterization and MRI features in a particular family, enables targeted genetic counselling on the avoidance of customary consanguineous marriage in such families.
